# Supplementary material for: Variation in soil properties under different cropping and other land-use systems in Dura catchment, Northern Ethiopia
Source: PLoS One. 2020 Feb 5;15(2):e0222476. doi: 10.1371/journal.pone.0222476 (PMC7001928; doi:10.1371/journal.pone.0222476)
Supplement: Attachment — Submitted filename: Response to Reviewers Comment-R1.doc [file pone.0222476.s002.doc]

**Journal: PLOS ONE

PONE-D-19-24034
Variation in Soil Properties under Long-Term Irrigated and Non-Irrigated Cropping and Other Land-Use Systems in Dura Catchment, Northern Ethiopia**


**Editor Comments**

1. **Comment:** In your Methods section, please provide additional location information of the sampling sites, including geographic coordinates for the data set if available.

**Reply:** Comment accepted and done accordingly (Page 7 lines 186-189).

2. **Comment:** In your Methods section, please provide additional information regarding the permits you obtained for the work. Please ensure you have included the full name of the authority that approved the sampling sites access and, if no permits were required, a brief statement explaining why.

   **Reply:** Included on page 12 lines 339-343.
3. **Additional Editor Comments (if provided):** Both reviewers have raised concerns regarding the fact that important information about the soils and the agricultural systems and practices have not been taken into account for the interpretation of your results, suggesting that irrigation might not be the most important factor explaining the differences in soil quality. I agree with them, so I would suggest to revise your work taking into account their comments in this direction.

   **Reply:** Revised accordingly (pls see the track changes in the paper)

**Reviewers' comments: Reviewer's Responses to Questions**

**Comments to the Author**

1. Is the manuscript technically sound, and do the data support the conclusions?

The manuscript must describe a technically sound piece of scientific research with data that supports the conclusions. Experiments must have been conducted rigorously, with appropriate controls, replication, and sample sizes. The conclusions must be drawn appropriately based on the data presented.

Reviewer #1: Partly

Reviewer #2: Partly

**Reply:** Revised to make the manuscript technically fully sound, and data support the conclusions (pls see marked paper)

2. Has the statistical analysis been performed appropriately and rigorously?

Reviewer #1: Yes

Reviewer #2: No

**Reply:** Addressed the concerns of Reviewer #2 as shown on the track changes (particularly pls see page 10 lines 264-265; and page 11 lines 293-298)

3. Have the authors made all data underlying the findings in their manuscript fully available?

   The [PLOS Data policy](http://www.plosone.org/static/policies.action#sharing) requires authors to make all data underlying the findings described in their manuscript fully available without restriction, with rare exception (please refer to the Data Availability Statement in the manuscript PDF file). The data should be provided as part of the manuscript or its supporting information, or deposited to a public repository. For example, in addition to summary statistics, the data points behind means, medians and variance measures should be available. If there are restrictions on publicly sharing data—e.g. participant privacy or use of data from a third party—those must be specified.

   Reviewer #1: Yes

   Reviewer #2: Yes

**Reply:** We have accepted the PLOS ONE policy

4. Is the manuscript presented in an intelligible fashion and written in standard English?

   PLOS ONE does not copyedit accepted manuscripts, so the language in submitted articles must be clear, correct, and unambiguous. Any typographical or grammatical errors should be corrected at revision, so please note any specific errors here.

   Reviewer #1: Yes

   Reviewer #2: Yes

**Reply:** Thanks, noted.

5. **Review Comments to the Author**


   **Reviewer #1:** This manuscript reports an attempt to identify key factors affecting soil quality (productivity) in a region where little is known about the interaction of soil properties and soil quality in small farmer agricultural production systems. Specific Comments are:

   **Comment 1.** Significant additional editing of English usage and grammar are needed throughout the manuscript.

**Reply:** Comment accepted and incorporated accordingly as it has shown as track changes.

**Comment 2.** Page 3, line 79: Change “have practiced” to “have been implemented”.

**Reply:** Comment incorporated on page 4 line 79.

**Comment 3.** Page 5, line 116: Change “to deal” to ‘dealing”.

**Reply:** Comment accepted (page 5 line 116)

**Comment 4.** Page 5, line 123: Insert “have” between “that” and “generalized”.

**Reply:** Comment accepted and done accordingly (page 5 line 124).

**Comment 5.** Page 5, lines 129-132: “…cannot be said…”? English editing???

**Reply:** Revised accordingly (page 5 line 131-132)

**Comment 6.** Page 5, lines 137-142: (Objectives) Are these properties to be related to specific land use/management practices? Is this information intended to be used only in the Dura sub-catchment or also used in other areas of Ethiopia? It might make this study more valuable in the authors could indicate if this concept is a “stand alone” effort for a small part of Ethiopia or whether it is part of a larger plan or concept for improving the agricultural lands in Ethiopia. Or, is it designed to specifically for education of small farmers. Placing this study into a larger context makes this work more valuable.

**Reply:** Done according to the comment (page 6 lines 141-142).

**Comment 7.** Page 6, line 170: What is an “irrigation command area”? Is it an irrigation project area? Is it an area where irrigated agriculture predominates? Is this an area where irrigation water is available but is used at the choice of the farmer? Is part of this study designed to evaluate the impacts of irrigation on soil quality outside of just making more water available for crops? An explanation of how this fits into the overall agricultural picture in the sub-catchment would provide a clearer context for the conduct of the study.

**Reply: Revised accordingly on page 7 line 172.**

**Comment 8.** Page 12, lines 328-336: It is not clear from the description of the sites what the actual soil types and parent materials exist at each sampled site. Differenced is sand can be due to erosion, deposition of erosion sediments or original soil parent material. The high clay component for the R1 and R2 systems are probably due to being in a more level depositional or alluvial landscape and may represent the Chromic Vertisols. Because they are nearly level and higher in clay, they are most suitable for irrigated agriculture. Care must be taken in interpreting data from a study like this because the properties of the soils may not be due to land use but rather the land use may be due to soil properties. A danger of this type of study is that it may be inappropriately interpreted if it is generalized over a larger area. The nature of soils in an area usually dictates how they are used. Also, use of fertilizer doesn’t usually affect soil texture. Inherent soil properties and erosion might be responsible, however. The fertilizer might have some effect on soil structure but not on soil texture.

**Reply:** Revised accordingly (page 7 lines 194-201; page 13 lines 355-362)

**Comment 9.** Page 12, lines 337-347: See the previous comment.

**Reply:** Revised accordingly (page 7 lines 194-201; page 13 lines 355-362)

**Comment 10.** Page 15, lines 429-445: The SAR of all of these soils in <1which means that Na is not a problem in the soil of the area, The authors indicate that the higher Ex Na in the TG is due to irrigation water runoff into the TG. However, water runoff into TG may or may not be responsible for the higher Ex Na. If it is, then the authors should show a link to the Na in the irrigation water (water quality?). It could also be due to the properties of the parent material.

**Reply:** Explanations included on page 16 lines 469-473.

**Comment 11.** Page 17, lines 507-509: Irrigation may also improve microbial activity because of more soil water conditions. This is difficult to interpret from C:N ratios. The C:N ratio of these soils are in the range of 10-12 which is quite normal for most soils. The TM (lowest) and TG (highest) may be the only systems that reflect the management systems but needs and examination of C and N inputs and erosion in these systems.

**Reply:** Explanations included on page 18 lines 551-557 during the revision.

**Comment 12.** Page 19, lines 563-566: This is difficult to interpret without knowing present and past erosion history.

**Reply:** Since sever erosion reports indicated from poor land use and management practices, it is possible to interpret it accordingly. Additional explanation has included on page 21 lines 617-620.

**Comment 13.** Page 19, line 580: Change “soil prosperities” to “soil properties”.

**Reply:** Done accordingly on page 21 line 633

**Comment 14.** Table 1: The rates of fertilizer need to be discussed more clearly either in the table or in the text. Potentially, information about whether DAP and urea were supplied annually, for each crop if double cropped or only occasionally within a cropping system is important in order to interpret the N and C status of the soils. “SWC practices were used at field borders” - What does this mean?

**Reply:** Comment accepted and described accordingly on Table 1.

**Comment 15.** Table 6: Generally in summary, the clay/CEC and SOM components explain most of the differences between the systems. Right? The first two PCA’s relate to the inherent soil properties and the last three relate to management. This again illustrates the need for knowledge of distinct soil types because these are mainly responsible for soil properties across management systems.

**Reply:** It could be for both the inherent soil properties and management practices. But in this study, it described that the differences in the component factors should be related to management practices and effect on erosion severity. Because even CEC and SOM are highly influenced by management practices and thereby the effects of SOM can be reflected on soil bulk density and soil N..

**Reviewer #2: General comments**

**Comment:** This work describes in detail the results of a survey conducted to identify contrasting agrosystems and other land uses in a small catchment in Northern Ethiopia, including irrigated and rainfed systems, a reference forest soil and eroded areas with and without reclamation measures.

In this sense, the study provides detailed information on the nine systems selected, both on terms of management and about soil physical and chemical properties of the studied upper layer (0-20 cm). It adds this valuable information to the existing knowledge on land uses in the region.

From these data, authors describe the major characteristics of the soils under these managements, and use PCA to depict those properties explaining better the observed variability, with the aim to identify useful soil quality indicators for further studies. This approach is interesting as it helps also summarizing the information obtained about the 28 soil properties studied.

From this, the study seems a good contribution to the knowledge of soil condition in the study area, and to the relationship between land uses and soil properties. From this perspective, it is therefore worth publication, although, to my view, there are some issues that would need to be addressed before acceptation:

As a major concern, the results of the different properties analyzed are studied as if the only factor possibly inducing differences was the type of soil management. This was however probably not the case, as significant differences were observed in relatively unchangeable soil properties such as texture. Accepting that some soil management conditions leading to intense erosion can result in textural changes at the topsoil (lines 330-332), the observed differences in clay content (from 73.7% in R2 to 26.3 under TM, Table 3) are very likely due to soil heterogeneity. Although the authors declare that all the plots selected were on the same type of soil (Chromic Vertisol), this is information is not clear enough to warrant similar soil characteristics in all plots. This seems therefore a study based in pseudo-replication, and this should be accounted for in the description of results and the discussion. As soil texture is determinant for many of the studied soil properties such as CEC, this heterogeneity imposes limitations to the comparisons among sites. This is acknowledged by the authors (line327) but not really considered in the discussion.

**Reply:** Revised accordingly with detail explanations on how management practices and erosion leads to textural variability in the same soil type (page 7 lines 194-201; page 13 lines 355-362)

**Comment:** In relation to the objective, although irrigation as a soil management system that can induce changes in soil condition is commented with some detail in the introduction, and included in the title, the study does not allow to draw clear conclusions on the effect of irrigation per se, as it compares irrigated and non-irrigated systems which differ in many other factors. For instance, the non irrigated systems (TM and MM) are monocultures, and the irrigated systems include intercropping and rotations, but not monoculture. Also MM and TM differ in the use of organic amendments in MM but not TM. Therefore, it seems more logical to compare the agrosystems between them (as such), but not trying to derive conclusions on the effect of irrigation as an isolated factor. My suggestion would be to remove from the title the idea that the study compares irrigated vs non-irrigated systems, and focus on the fact that it makes a comprehensive revision of the most common agrosystems and land condition (forest, gullies) found in the region.

**Reply:** Comment accepted and revised accordingly starting from the title. But attention was given on the two agricultural practices which are irrigation and non irrigation (rain-fed) to show the roles of such bold activities in the CLUS.

**Comment:** Some of the properties studied, and in particular aggregate stability (but also SAR and porosity for instance) were not directly analyzed, but derived from others in the dataset. This needs to be handled with care when studying correlation in the PCA, as of course variables calculated from other variables would correlate with them. In particular, the formula used to calculate aggregate stability needs to be revised (line 248): as it is written, under equal SOC concentrations, this expression would result in a lower value (less stability) for a soil with more clay and silt (less sandy) than to a sandy soil with less clay + silt. This is counterintuitive and probably the cause of some unexpected results in this parameter found in Table 3.

Reply: Comment accepted and the formulas are revised to calculate with respect to each CLUS (page 10 line 265, and 277

**Comment:** The calculation of SOC stocks for comparing (supposedly) the same soil under different management practices needs an approach based in comparing equivalent masses (i.e., the amount of SOC in the same mass of soil for all managements considered). Although the reference used to describe the methodology for SOC storage does in fact describe this point (Ellert & Bettany, 1995), the authors did not use this approach. As differences were observed in bulk density, using the same formula provided in line 274 for two soils with the same SOC concentration but different bulk density would result in a greater stock in the one with higher bulk density. To avoid this bias, the approach of an equivalent mass comparison requires some easy calculations to correct the depth (T) considered in each case. As a summary, my recommendation is that the authors consider these aspects and run the comparative study with the new perspective and data, including PCA. I hope that these comments, which are meant to be constructive, are of use for a fruitful revision of the manuscript.

**Reply:** Comment accepted and revised accordingly on page 10 lines 290-291; page 11 lines 292-298

**Some other minor comments are:**

**Introduction:**

**Comment:** L.75-76: Irrigation can indeed increase SOC by increasing primary productivity, but contrasting results have been reported in this sense (see for instance recently daGama et al., 2019. Agronomy 2019, 9, 132; doi:10.3390/agronomy9030132).

**Reply:** Correct. If irrigation causes salinity related problems the practice can decrease SOC. However, in the conditions of the study area where salinity is not a problem, irrigation can increase SOC by improving biomass production. This has explained on page 3 lines 75-76.

**Comment:** L.103-109: This paragraph would be better in the paragraph in lines 61-62, as it contains very similar information.

**Reply:** Comment accepted and included on page 4 lines 92-98.

**Comment:** L.82. Soil organic C is a nutrient? For what organisms? For plants?

**Reply:** Explanation included on page 4 and lines 82-83.

**Comment:** L.137-142. See previous comment on the problems to compare irrigation as a factor in this study in relation to objective one. Suggest to replace by “under different cropping and other land-use systems”.

**Reply:** Comment accepted and revised accordingly starting from the title page and through out the paper. But there are conditions to mention irrigation and non-irrigated practices.

**Materials and methods**

**Comment:** L.162. Same comment on irrigated and non-irrigated. Why stressing this and not for instance adjacent mono and poli-culture systems?

**Reply:** Comment accepted and revised accordingly (Page 6 lines 164-166.

**Comment:** L.209-211. The reader has to rely about the homogeneity of the different areas based on this statement. However, results (Table 3) indicate that soils were different at least in one genetic characteristic such as topsoil texture... This should be considered in the discussion.

**Reply:** Comment accepted (Page 7 lines 196-201; page 8 lines 202-203); and page 13 lines 356-364.

**Comment:** L.226 no need to indicate irrigated and non-irrigated, as undisturbed soil samples were also collected from the native forest and the unreclaimed gully for instance...

**Reply:** Corrected page 9 line 245.

**Comment:** L.247-254. See previous comment on this index for soil structural stability. If this parameter is not to be measured directly on soil samples, to my view, better formulas are available in the literature to relate texture and organic matter to aggregate stability, that consider the role of BOTH organic matter and soil clays in structure stabilization.

**Reply:** There may be other formulas, but the existing formula is suggested in different sources to be used to assess soil structure stability in which this is also adopted to the study area conditions.

**Comment:** L.272-280. See comments above on the use of equivalent masses to compare SOC (and TN) stocks.

**Reply:** Comment accepted and incorporated as shown on page 11 lines 292-300.

**Comment:** L.285-289. Without commenting on the adequateness of ANOVA for this treatment, management systems are used here as one factor, without separating “irrigation” as an independent factor. This supports my previous observations that irrigation should not be commented in this work as a factor of study. It just makes part of some of the agrosystems studied, and it can be used to explain the results, just as organic fertilization, afforestation, tillage and the other parameters included in the management routines of the agrosystems described in Table 1.

**Reply:** Comment accepted and ANOVA was used a factor of the different CLUS. Irrigation and non irrigation was considered as part of the practices in the CLUS.

**Results & Discussion**

**Comment:** L.322-327. See comments above on the difference in texture. It seems unlikely (and cannot be proved with this study) that soil management is the responsible of all the differences observed in clay content among the nine studied sites.
**Reply:** Comment accepted (Page 7 lines 196-201; page 8 lines 202-203); and page 13 lines 356-364.

**Comment:** L.390-391. Why should irrigation increase the soil pH? Is there anything in irrigation water quality that suggests so? Why it would only affect R1 and not the other irrigated treatments?

**Reply:** Explanation included on page 15 lines 423-426.

**Comment:** L.421-422. What is the relationship between soil management and CEC?

**Reply:** Explanation is shown on page 16 lines 460-462

**Comment:** L.429-445. Some discussion is done here on NA and SAR. I agree that the values reported are not within the range of sodicity, but could the different values observed between treatments affect for instance soil aggregation and therefore infiltration, clogging, etc?
Reply: Explanations included on page 16 lines 471-476; and page 17 lines 488 – 491.

**Comment:** L.505. So we discover here that the “non-irrigated” TM treatment is water-saturated more than 6 months per year. How can this have changed soil properties other than SOC?

**Reply:** Explanation included on page 19 lines 553-556.

**Comment:** L.534-576. See comments above on the comparisons on SOC that require equivalent mass corrections.

**Reply:** Comment accepted and incorporated as shown on page 11 lines 292-300.

**Comment:** L.580 Soil prosperities? (guess properties...)

Reply: Corrected on page 21 line 636.

**Comment:** L. 596-623. See previous comment on covariance and correlation of variables derived mathematically from each other.

**Reply:** Revised accordingly to reflect each CLUS.

**Conclusions**

**Comment.** Can be revised after addressing previous comments.

**Reply:** Revised accordingly (pls see on page 24)
