# Supplementary material for: Variation in soil properties under different cropping and other land-use systems in Dura catchment, Northern Ethiopia
Source: PLoS One. 2020 Feb 5;15(2):e0222476. doi: 10.1371/journal.pone.0222476 (PMC7001928; doi:10.1371/journal.pone.0222476)
Supplement: Attachment — Submitted filename: Response to Reviewers Comments-PLOS ONE-R2.docx [file pone.0222476.s003.docx]

**Journal: PLOS ONE**

PONE-D-19-24034R1
Variation in soil properties under different cropping and other land-use systems in Dura catchment, Northern Ethiopia


**Editor Comments**

**Comment:** Both reviewers agree that the modifications performed have improved the manuscript, but they still feel that several changes must be made before publication. In addition to their specific comments, it should be more clearly acknowledged that the soil variability observed in your study could be due to intrinsic soil heterogeneity in such a wide area, and not only to cropping system or land-use.
**Reply:** Yes, of course, that is why the PCA determinant soil parameters explain the soil quality variability by the CLUS by 87%, indicating the rest 13% could be intrinsic factors (page 24 line 709).

**Reviewer's Responses to Questions**

**Comments to the Author**

1. If the authors have adequately addressed your comments raised in a previous round of review and you feel that this manuscript is now acceptable for publication, you may indicate that here to bypass the “Comments to the Author” section, enter your conflict of interest statement in the “Confidential to Editor” section, and submit your "Accept" recommendation.

Reviewer #1: All comments have been addressed

Reviewer #2: (No Response)
**Reply with reference to Reviewer #2:** In this revision, your comments were well addressed. Pls see the track changes in the paper.

2. Is the manuscript technically sound, and do the data support the conclusions?

The manuscript must describe a technically sound piece of scientific research with data that supports the conclusions. Experiments must have been conducted rigorously, with appropriate controls, replication, and sample sizes. The conclusions must be drawn appropriately based on the data presented.

Reviewer #1: Yes

Reviewer #2: Partly

**Reply with reference to Reviewer #2:** Pls seethe revised conclusion on page 26.

3. Has the statistical analysis been performed appropriately and rigorously?

Reviewer #1: Yes

Reviewer #2: No

**Reply with reference to Reviewer #2:**   Revised paper with due attention to the calculation of soil mass, equivalent soil mass and the OC and TN stocks as indicated on page 11 lines 299-318; page 12 lines 319-342; and Table 5.

4. Have the authors made all data underlying the findings in their manuscript fully available?

The [PLOS Data policy](http://www.plosone.org/static/policies.action#sharing) requires authors to make all data underlying the findings described in their manuscript fully available without restriction, with rare exception (please refer to the Data Availability Statement in the manuscript PDF file). The data should be provided as part of the manuscript or its supporting information, or deposited to a public repository. For example, in addition to summary statistics, the data points behind means, medians and variance measures should be available. If there are restrictions on publicly sharing data—e.g. participant privacy or use of data from a third party—those must be specified.

Reviewer #1: Yes

Reviewer #2: Yes

5. Is the manuscript presented in an intelligible fashion and written in standard English?

PLOS ONE does not copyedit accepted manuscripts, so the language in submitted articles must be clear, correct, and unambiguous. Any typographical or grammatical errors should be corrected at revision, so please note any specific errors here.

Reviewer #1: Yes

Reviewer #2: Yes

6. Review Comments to the Author

Please use the space provided to explain your answers to the questions above. You may also include additional comments for the author, including concerns about dual publication, research ethics, or publication ethics. (Please upload your review as an attachment if it exceeds 20,000 characters)

**Reviewer #1:** The authors have satisfied most of this reviewer’s concerns. However, a few concerns still remain:

**Comment:** 1. Pages 19-20, lines 571-610: Change “OC:TN” ratio to “C:N” ratio. This is the most commonly used form and is understood by most soil scientists and agronomists.

**Reply:** Comment accepted and done accordingly (Page 11 lines 292-297 ).

**Comment:** 2. Page 20, lines 581-591: The authors seem to place a great deal of focus on soil C:N ratios. Soil C:N ratios are a function of quality of inputs minus the outputs of soil biological systems. The C:N ratios of these soils range from 9.6 to 13.7. These values reflect the “balance” of the inputs (C and N materials) vs. the outputs (CO2 and mineral N to the crops or leaching) as mediated by soil microorganisms. These value generally lie in the normal range of 10-12 (mean=11.8) with the outliers very close to this range. The extreme of 9.6 (TM) may reflect the annual N fertilization that might be greater than the crop needs. The other extreme of 13.7 (TG) may reflect no outside fertilization and the natural biomass inputs from the grasses growing on the land. The leguminous trees may or may not have much impact on the soil C:N status. Unless there are soil samplings at or near fertilizer or plant residue applications, the C:N ratios appear to be in “balance” with each system. When C:N ratios are near normal, then minor differences in C:N ratio will have very little effect on soil productivity because other productivity factors (such as water availability) will override any fertility influenced by the soil C:N ratio.

**Reply:** Revision was made considering the comment above (Page 20 line 594; page 21 lines 612-615 ). However, with regard to the C:N ratios range (10-12) commented as the ratios lie in the normal range, but explanations are available on page 21 lines 612-615 as it indicates that C:N ratio less than 10 is good (normal) soil condition.

**Comment:** 3. Additional English editing is still needed.

**Reply:** Editing was done accordingly (see throughout the paper for marked changes)

**Decision of reviewer #1:** This manuscript should be accepted for publication after minor revision based on the above comments. This is an interesting study that is useful for farm managers in many regions of the globe.

**Reviewer #2: PONE-D-19-24034.R1**
Variation in soil properties under different cropping and other land-use systems in Dura catchment, Northern Ethiopia

**General comments**
This manuscript is a revised version of a work I revised before. I stand to my previous comments that the study is of interest, mostly because it adds comprehensive information about soil parameters in an area where this is scarce. It also offers an overview of soil variability in the region, and in relation to cropping and land-use systems (CLUS). I appreciate the approach based in participation of farmers in the selection of CLUS and representative plots.
In my previous report, I addressed however some points of concern in relation to the approach, some methodologies, and, in particular, the impossibility of deriving conclusions related only to the effect of CLUS in the region from the sampling scheme designed and conducted in the study.
I appreciate the efforts done by the authors to modify and improve the manuscript to this respect. Still, in addition to some methodological considerations (see below), which, to my view are not still clear and could be improved, there is still in the manuscript a view that implies establishing a cause-effect relationship between CLUS and soil condition (soil parameters). Although this is very likely the case for many properties, it is still difficult to be completely assertive (as authors are) in this respect considering that o**nly one plot per CLUS** was sampled in the whole catchment area, and that no clear information on soil natural heterogeneity is provided.

**Reply:** Three plots per CLUS was used as this is indicated on page 10 lines 233-235.

Comment: For instance, authors (lines 194-201) assign the **observed (very high) variability** in topsoil texture to land management. The explanations provided seem reasonable, but still this is more an **observational information** than evidence-based data. Also, they acknowledge later (l.355) that such a heterogeneity “could influence the other textural classes and physical and chemical soil properties”. If so, the question remains open on whether it is soil characteristics which induce the particular selection of one use, or if it is the use which results in the observed status of soil parameters.
With this (and the comments below), my view is that the manuscript needs to be revised (**mostly in the discussion**) to account for this particular issue.

**Reply-a:** With regard to the observation information which is commented as it is not evidence-based data, it is quiet write to have evidence-based data. However, to have such data it demands additional research which is not in line with the purpose of the present paper. Rather our explanation was relied on the logical information collected from the farmers discussion and field observation that states that there is long-term variability in management practices and cropping systems in which such variation could lead to differences in soil properties including soil texture (Page 7 lines 173-197). In addition, it is indicated that the soil properties variability is mainly related to the CLUS (87%), but the rest 13% may be intrinsic factor (Page 24 lines 709-710 ).

**Reply-b:** With regard to the comment “Also, they acknowledge later (l.355) that such a heterogeneity “could influence the other textural classes and physical and chemical soil properties”. If so, the question remains open on whether it is soil characteristics which induce the particular selection of one use, or if it is the use which results in the observed status of soil parameters.”
The **reply** to this comment is that “the sentences …influence the other textural classes and physical and chemical soil properties” is deleted during this revision in order to reduce confusion by the readers so that to make clear the cause for the soil properties variability in the study area is related to the different CLUS and their corresponding soil mgt practices (page 14 lines 397-398).

**Specific Comment:** In addition, and also related to my previous comments, some methodological aspects that would need some clarification are:
**Comment 1-** The calculation of SOC stocks using an equivalent mass approach needs, as a first step, to determine such equivalent mass. As the formulae provided are not clear to this respect, and data of bulk density and SOC and NT stocks provided in Table 5 show clear differences among CLUS (Table 3), I believe it would be easier for readers to provide the **value of the equivalent mass chosen** in the M&M section, when the method is explained, and then provide SOC and TN stocks data in relation to that mass.

**Reply:** Comment accepted and revised accordingly (Page 11 lines 300-319; page 12 lines 320-343) and also Table 5.

**Comment 2-** The pedo-transfer equation used for aggregate stability has not changed since the first version. Authors argue that it has been used in other studies and therefore validated by evidence. Since it still implies that same SOC values in a soil with less clay would result in lower aggregate stability than in a soil with more clay, I’d suggest that the authors provide at least the references they mention to support its use.

**Reply:** Revision Done accordingly (page 10 line 277-282). Plus see page 16 lines 455-457.

**Comment 3-** The PCA has not changed since the original version, nor its discussion. Still, I believe some comments are needed on the use of derived properties such as aggregate stability, which generate auto-correlation based in calculations, not measured data.

**Reply:** Yes of course, on the concern of auto-correlation, but it was handle by partial correlation analysis and also excluded from the PC considering the correlation and loadings as indicated on page 24 lines 701-705; 722-725; page 25 lines 737-750)

I sincerely thank you the editor and the anonymous reviewers for the constructive comments and suggestion.

**The End!**
